# Dynamic crystal rotation resolved by high-speed synchrotron X-ray Laue diffraction

**DOI:** 10.1107/S160057751600223X

**Published:** 2016-03-30

**Authors:** J. W. Huang, J. C. E, J. Y. Huang, T. Sun, K. Fezzaa, S. N. Luo

**Affiliations:** a The Peac Institute of Multiscale Sciences, Chengdu, Sichuan 610031, People’s Republic of China; b Key Laboratory of Advanced Technologies of Materials, Ministry of Education, Southwest Jiaotong University, Chengdu, Sichuan 610031, People’s Republic of China; c Advanced Photon Source, Argonne National Laboratory, Argonne, IL 60439, USA

**Keywords:** synchrotron X-ray Laue diffraction, crystal rotation

## Abstract

Dynamic compression experiments are performed on single-crystal Si under split Hopkinson pressure bar loading, together with simultaneous high-speed (250–350 ns resolution) synchrotron X-ray Laue diffraction and phase-contrast imaging. A methodology is presented which determines crystal rotation parameters, *i.e.* instantaneous rotation axes and angles, from two unindexed Laue diffraction spots. Two-dimensional translation is obtained from dynamic imaging by a single camera. High-speed motion of crystals, including translation and rotation, can be tracked in real time *via* simultaneous imaging and diffraction.

## Abstract 2

High-speed (submicrosecond) translation and rotation of crystals are tracked in real time *via* simultaneous synchrotron X-ray imaging and Laue diffraction.

## 1. Introduction   

Grain or crystal rotation is a key mechanism of accommodating elastic or plastic deformation in crystalline solids (Chen *et al.*, 2013 ▸). In quasi-static experiments, crystal rotation is normally resolved with electron backscattering diffraction (Zaafarani *et al.*, 2008 ▸) or X-ray Laue diffraction (Maaß *et al.*, 2007 ▸, 2008 ▸). Single-shot multiframe *in situ* real-time measurements on crystal rotation during dynamic loading have been an experimental challenge, especially for high strain rate loading in the nano- to micro-second regimes with a split Hopkinson pressure bar (SHPB) (Kolsky, 1949 ▸) or a gas gun (Fowles *et al.*, 1970 ▸). In powder technology and fluid mechanics, rotation of particles or granules plays a significant role in the gas–solid interactions, including two-phase flows, heat transfer and coal combustion (Kajishima, 2004 ▸; Goldschmidt *et al.*, 2004 ▸; Sun & Battaglia, 2006 ▸). Detection of high-speed particle rotation is in urgent need. For example, Wu *et al.* (2008 ▸) used optical imaging to measure particle rotation at about 500 revolutions per second (r s^−1^) in a circulating fluidized bed. X-ray diffraction is a useful complement for rotation measurements given its penetration capability and higher resolution. X-ray diffraction was used to study the quasi-static packing and deformation characteristics of sands (Hall *et al.*, 2011 ▸). Other applications may involve measuring remotely translation and rotation of a fast-moving object.

Synchrotron X-ray Laue diffraction possesses unique advantages for lattice-level measurements under dynamic loading and has been under intensive development along with synchrotron X-ray imaging (Luo *et al.*, 2012 ▸; Hudspeth *et al.*, 2013 ▸; Lambert *et al.*, 2014 ▸; Turneaure *et al.*, 2009 ▸; Wei *et al.*, 2004 ▸) for studying high-strain-rate deformation, fracture and phase change of a variety of materials. In particular, a real-time *in situ* simultaneous X-ray imaging and diffraction technique has been demonstrated recently at the Advanced Photon Source (Fan *et al.*, 2014 ▸; Hudspeth *et al.*, 2015 ▸). In this work, we choose single-crystal Si, brittle and of high modulus, for dynamic loading with a SHPB, and perform simultaneous X-ray imaging and Laue diffraction, in order to establish an illustrative case for quantitative rotation and translation measurements. A theoretical procedure is introduced to resolve three-dimensional high-speed rotation of a single crystal with two unindexed diffraction spots. Three-dimensional crystal rotation at approximately 300 r s^−1^ is resolved. High-speed motion of crystals, including two-dimensional translation and three-dimensional rotation, can be tracked in real time from simultaneous imaging and diffraction.

## 2. Methodology   

**Figure 1:**
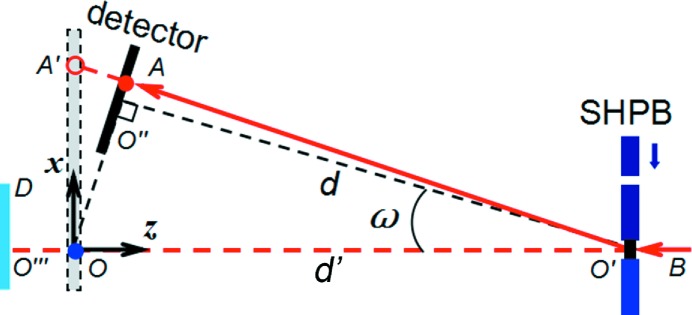
Experimental setup for dynamic X-ray diffraction with simultaneous imaging and the coordinate system (*O* *xyz*). Images form on the 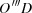 plane along the 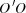 direction. 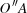 is the diffraction detector plane. 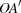 is the virtual detector for diffraction. SHPB: split Hopkinson pressure bar (loading device); 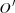: sample position; 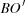: incident X-rays; 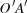: diffracted X-rays; 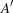: a diffraction spot on the virtual detector (the *xy* plane). Also see text.

The experimental methodology for simultaneous synchrotron X-ray imaging and diffraction has been established at beamline 32-ID-B of the Advanced Photon Source (Fan *et al.*, 2014 ▸; Hudspeth *et al.*, 2015 ▸). The schematic setup for simultaneous diffraction and phase contrast imaging is shown in Fig. 1 ▸, along with the coordinate system. We perform high-strain-rate loading on a brittle Si single-crystal with SHPB. Prior to loading, the crystallographic directions [0
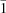
1], [2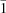 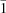] and [111] are oriented approximately along the *x*, *y* and *z* directions, respectively. Dimensions of the sample perpendicular to the X-ray direction (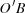) are 3 mm × 3 mm and the thickness along the X-ray direction is 1 mm. After the gas gun of the SHPB is fired, a compression pulse is imposed on the sample. A tensile pulse ensues upon reflection between the bar–sample interface, and the incident bar is then separated from the sample. The sample retains its overall integrity and is subjected to rigid-body motion, including translation and rotation, when a stress is below the fracture strength of Si (∼1 GPa).

We perform simultaneous X-ray imaging and diffraction measurements on the sample. The probe X-rays are from an APS ‘undulator A’ light source and the undulator gap is 25 mm. The spectral flux–photon energy curves of the undulator source are presented elsewhere (Luo *et al.*, 2012 ▸). The probe X-rays (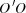) illuminate the sample perpendicular to the loading direction. The transmitted X-rays are collected by an imaging scintillator, Ce-doped Lu_3_Al_5_O_12_ (LuAG:Ce), and recorded with a Photron FastCam SA-Z (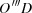). The exposure time is 0.35 µs and the frame interval is 10 µs. At the same time, the scattered light is collected by a diffraction scintillator, Ce-doped Lu_2–2*x*_Y_2*x*_SiO_5_ (LYSO:Ce), and is recorded with another SA-Z after passing a microchannel plate (MCP, Quantum Leap E, Stanford Computer Optics, Inc.), synchronized with the imaging camera. The exposure time is 0.25 µs and the frame interval is 10 µs. The number of activated pixels is 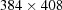. The diffraction camera is mounted on a rotation stage for continuous rotation in the circumferential direction. A Huber 410 goniometer is used to control the rotation stage with a resolution of 0.001°. The scintillator, MCP and SA-Z together are equivalent to an area detector (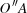). The pixel size is calibrated to be 60 µm × 60 µm for the current geometry. The sample-to-diffraction detector distance (*d*) is 230 mm and the angle 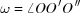 between the incident beam and the normal of the diffraction detector plane is 24.5°.

Each Laue diffraction spot corresponds to lattice planes with certain spatial orientation. Lattice deformation and crystal rotation can both contribute to its shift. Laue diffraction pattern analysis has been widely applied to obtain strain tensor (Li *et al.*, 2015 ▸; Liu *et al.*, 2014 ▸; Wang *et al.*, 2011 ▸; Barabash *et al.*, 2010 ▸) and grain rotation (Maaß *et al.*, 2007 ▸, 2008 ▸); there are many dedicated software packages available for this analysis (Tamura, 2014 ▸; Micha & Robach, 2014 ▸; Huang, 2010 ▸). A method based on digital image correlation using Laue spots as speckles has been developed recently for strain analysis (Borbély, 2015 ▸). However, the effective area of detectors available for dynamic events is very limited and the sample–detector distance has to be sufficiently large in order to accommodate the imaging camera as well as protective components for impact loading. As a result, the number of recorded diffraction spots is small (*e.g.* 1 or 2). Meanwhile, a conventional Hopkinson bar setup is not intended for high-accuracy alignment as required for Laue diffraction indexing. So indexing the spots and orienting a crystal are currently impractical, and the aforementioned Laue analysis tools are not applicable. In our experiments, the sample moves stress-free, so the remaining lattice strain after low-speed impact is small and its contribution to diffraction spot movement is negligible compared with rotation. Therefore, we establish a quantitative relationship between three-dimensional crystal rotation and two-dimensional movement of two or more diffraction spots, which is elaborated in Appendix *A* [App appa]. In this method, neither crystal orientation nor indices of diffraction spots are needed to obtain rotation parameters (rotation axes and rotation angles).

## 3. Results and discussion   

**Figure 2:**
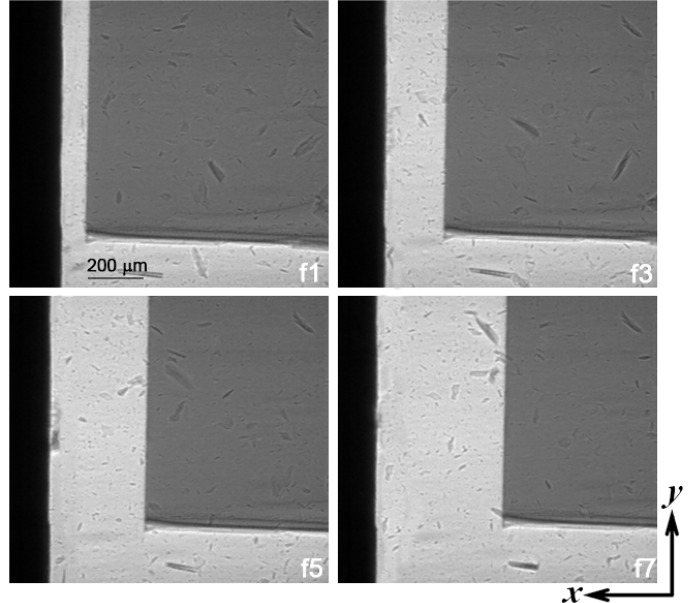
Selected phase-contrast images of the sample obtained simultaneously with diffraction shown in Fig. 3 ▸. The black stripes on the left refer to the recoiling steel bar after impact. f*N* denotes the *N*th frame. The frame interval is 10 µs. Loading direction is from left to right.

The dynamic loading experiment was conducted at a projectile velocity of 20 m s^−1^. Results from simultaneous imaging and diffraction are presented in Figs. 2 ▸ and 3 ▸.

During recoil, the incident bar separates from the Si sample at 1 m s^−1^. The sample remains essentially intact except that minor fragments form from stress concentrations during impact and flies in free rigid-body motion along the loading direction (Fig. 2 ▸). The two-dimensional phase-contrast images supply information on planar translation and rotation of the sample, but not on out-of-plane motion. The translation velocities are approximately 5 m s^−1^ and 1 m s^−1^ in the *x* and *y* directions, respectively, and the in-plane rotation speed is small.

**Figure 3:**
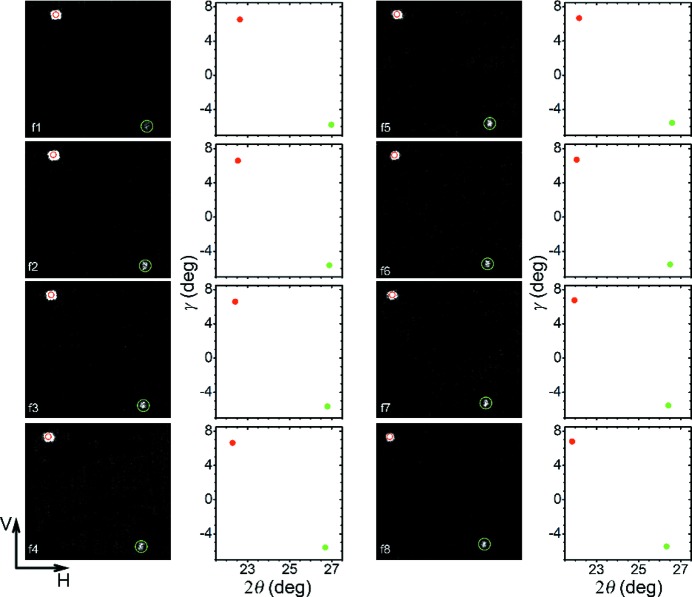
Time-resolved synchrotron X-ray diffraction. Columns 1 and 3 are two-dimensional diffraction patterns, and columns 2 and 4 are the corresponding 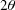
–γ plots (filled circles). Empty circles denote the diffraction spots predicted from the rotation parameters. V: vertical; H: horizontal.

Eight representative frames of diffraction patterns containing two diffraction spots are chosen for analysis (Fig. 3 ▸, columns 1 and 3). Two diffraction spots shift simultaneously from the lower right to upper left of the screen, without change in spot shape. This shift is attributed to sample or crystal rotation, rather than lattice compression or tension, because the sample has already been subjected to unloading and detached from the incident bar during the time window of interest here. The minute residual lattice deformation (Noyan & Cohen, 2013 ▸) cannot induce such a pronounced shift. Note that there is only one diffraction spot recorded prior to frame 1 (Fig. 3 ▸). As the sample undergoes rotation during impact (before frame 1), indexing the spots before and after frame 1 is not possible even though its initial orientation is known. However, a quantitative analysis of crystal rotation can be conducted from two Laue spots with the scheme detailed in Appendix *A* [App appa].

As each harmonic of the undulator radiation has a finite bandwidth (∼2% for the APS undulator A light source) (Fan *et al.*, 2014 ▸), some diffraction spots do not vanish from the field of view during rotation, within the time window of observation. As discussed above, relative translation and rotation of the sample between adjacent frames on the *Oxy* plane is tiny due to short frame intervals (Fig. 3 ▸), so the small-angle assumption in equation (8) of Appendix *A* [App appa] is reasonable.

Each pixel on the diffraction detector is mapped onto the 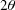
–γ plane. Given the finite size of a diffraction spot, we use Gaussian fitting to obtain the coordinates of its center (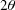, γ). The diffraction spots are plotted on the 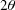
–γ plane in columns 2 and 4 of Fig. 3 ▸. Then, the relative rotation axes (expressed in three projection quantities) and the rotation angles between adjacent frames are calculated (Table 1 ▸). The rotation directions during the free motion of the sample appear to be random. The rotation angles are extremely small between two neighboring frames (0.05–0.13°), consistent with the small-angle assumption. The sample mainly undergoes translation along the loading (*x*) direction with concurrent, small and irregular rotation. The high angular resolution of dynamic Laue diffraction is advantageous for such applications as determining grain rotation in a polycrystalline solid.

As a self-consistency check, we conduct forward calculations using the rotation parameters in Table 1 ▸ to compute the diffraction spot positions on the diffraction detector. Given the rotation axes and angles as well as the initial positions of diffraction spots, their positions at any instant can be obtained as elaborated in Appendix *A* [App appa]. The simulated diffraction spot positions are listed in Table 2 ▸ and plotted as empty circles in columns 1 and 3 of Fig. 3 ▸. The comparison shows a complete coincidence between the simulations and the measurement.

**Figure 4:**
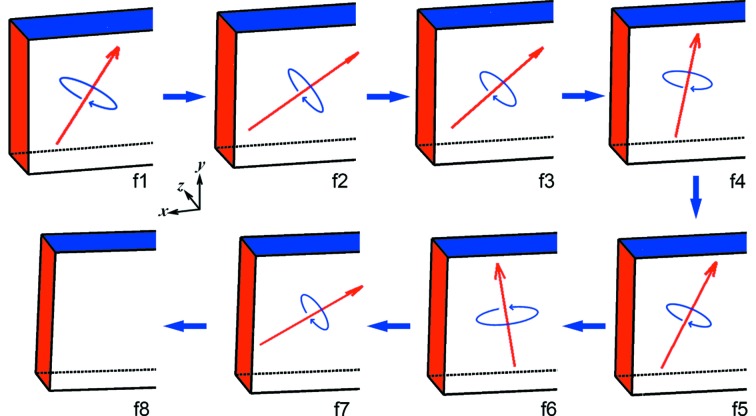
Illustrations of the evolution of sample position and orientation, corresponding to Figs. 2 ▸ and 3 ▸. The rotation angles are magnified by ten for clarity. Red arrows: rotation axis; blue elliptic arrows: rotation direction.

The two-dimensional translation and full rotation parameters as a function of time (Table 1 ▸) are illustrated in Fig. 4 ▸, using a schematic object. Therefore, rigid-body motion, including translation and three-dimensional rotation, of the sample can be strictly mapped through simultaneous two-dimensional X-ray imaging and diffraction. Translation mapping can be extended into three dimensions if an extra camera is used at a different view angle. Simultaneous high-speed tracking of translation and rotation in three dimensions offers great potential in a multitude of applications.

We present the simplest case in the above discussion. For strong shocks, both lattice strain and grain rotation contribute to the movement of a diffraction spot. Strain can be evaluated from conventional methods such as known equation of state, and it is then decoupled from rotation to a certain extent. However, more diffraction spots are certainly desirable.

## 4. Conclusion   

We have performed high-speed simultaneous synchrotron X-ray Laue diffraction and phase-contrast imaging measurements on single-crystal Si under SHPB loading, and developed a methodology for quantifying rotation parameters, *i.e.* rotation axis and rotation angles. The exposure time is 250–350 ns and the frame interval is 10 µs. High-speed crystal rotation at about 300 r s^−1^ is resolved. High-speed motion of crystals, including two-dimensional translation and three-dimensional rotation, can be tracked in real time from simultaneous imaging and diffraction.

## Figures and Tables

**Figure 5:**
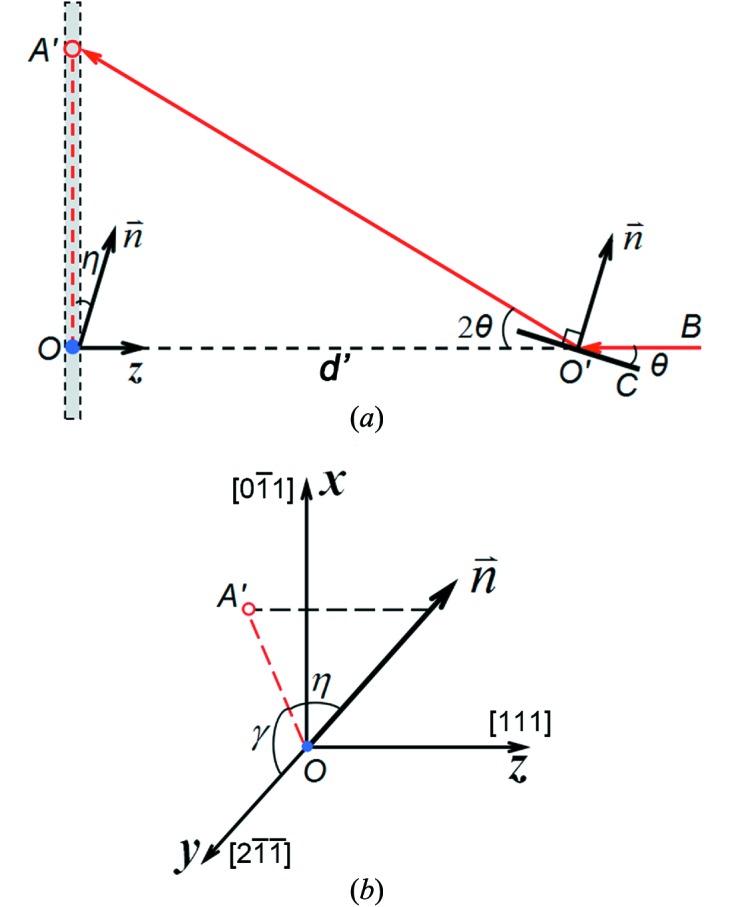
(*a*) Diffraction geometry. (*b*) Definitions of lattice plane normal **n**, and angles of γ and η. Symbols are the same as those in Fig. 1. ▸

**Table 1:** Rotation parameters at the instants noted in Fig. 3 ▸

	Rotation axis			
Frame	*x*	*y*	*z*	Rotation angle (°)
1–2	−0.5551	0.4298	0.7121	0.1149
2–3	−0.7023	0.7039	−0.1065	0.0744
3–4	−0.7398	0.5853	0.3319	0.0988
4–5	−0.2884	0.8321	0.4737	0.0626
5–6	−0.4736	0.7842	0.4010	0.0689
6–7	0.0135	0.8960	0.4439	0.0523
7–8	−0.8573	0.3840	0.3429	0.1262

**Table 2:** Measured and simulated positions of the two diffraction spots on the detector V and H denote vertical and horizontal positions in pixels, respectively. Subscripts 1 and 2 denote Laue diffraction spots 1 and 2, respectively.

	Experimental				Simulated			
Frame	V_1_	H_1_	V_2_	H_2_	V_1_	H_1_	V_2_	H_2_
1	86	40	341	353	86	40	341	353
2	81	39	335	348	80	38	336	349
3	73	40	330	348	73	39	331	349
4	66	40	324	346	65	39	325	347
5	59	39	319	345	59	39	319	345
6	53	39	313	343	52	39	313	344
7	46	39	308	343	46	39	308	342
8	39	39	303	340	39	39	304	340

## Appendix A Determining rotation parameters from two Laue diffraction spots

The geometry of diffraction is defined in Figs. 5 ▸(*a*) and 5(*b*). Incident X-rays () are scattered by lattice planes () and the scattered X-rays () yield a diffraction spot () on a diffraction detector () located at an arbitrary position. The origin, *O*, is the transmission spot on the detector plane of the incident X-ray beam propagating along the −*z* direction. For convenience, we introduce the virtual detector plane containing *O*, *i.e.* the *Oxy* plane, perpendicular to the incident X-ray beam. A diffraction spot () on the detector is mapped onto the virtual detector plane or *vice versa*. Discussions refer to the virtual detector plane () unless stated otherwise.

The diffraction angle  and . The azimuthal angle of a diffraction spot, γ, is the angle formed between  and the *x* axis. The distance between the sample and the origin is . In terms of  and the mapped coordinates  of a diffraction spot:

The unit vector **n** is the normal of certain parallel lattice planes. The angle between **n** and  is defined as η, and . Therefore,

**n** is expressed in terms of γ and θ obtained from two-dimensional diffraction patterns, so the instantaneous orientation of the lattice planes is determined.

The rotation axis and rotation angle are chosen to describe sample rotation, and are denoted by a unit vector  and α, respectively. Here, superscript T denotes the transpose. The corresponding rotation matrix  can be expressed in terms of **u** and α as

After sample rotation, instantaneous normal vector  at time  changes to  =  at . The rotation matrix, , relates  and  *via*

The objective is to solve  and α from equation (7), which fully characterizes the rigid-body rotation of the sample. This equation can be solved numerically. For small rotation angles, it can be simplified with  and . Equation (7) is then reduced to

Subtracting  on both sides of equation (8) yields

where ,  and .

For convenience, the Euler vector **E** is defined as

It follows that

There are three unknowns, , , . But these three equations are linearly dependent or contradictory. Thus, in order to determine , another diffraction spot is needed. Given two diffraction spots from different sets of lattice planes, this problem becomes overdetermined (six equations *versus* three unknowns) and the rotation parameters can be fully solved as

Note that even more diffraction spots can be used and the uncertainties are reduced.

For each diffraction spot, a set of  and thus a set of  are obtained. Two independent spots yield two sets of . The rotation parameters, α and **u**, are solved from four diffraction spots for frames 1 and 2. Repeating such a process between two adjacent frames yields the rotation history of the sample. A MATLAB code is developed to perform such analysis in a batch mode.

To validate the above derivations, we perform forward analysis, *i.e.* calculate the diffraction spots on the detector from rotation parameters α and **u** obtained from the diffraction spots of frames 1 and 2 as described above.  is calculated from the initial positions of a diffraction spot at frame 1. After rotation,  changes to , which is calculated with the Rodrigues’ rotation formula (Rodrigues, 1840 ▸)

Then,

On the virtual detector plane,

The simulated diffraction spot  is then mapped onto the detector and should coincide with the directly measured spot at frame 2.
